# Effects of Income and Material Deprivation on Children’s Life Satisfaction: Evidence from Longitudinal Data for England (2009–2018)

**DOI:** 10.1007/s10902-021-00457-3

**Published:** 2021-10-05

**Authors:** Gundi Knies

**Affiliations:** Johann Heinrich von Thünen Institute, Braunschweig, Germany

**Keywords:** Children, Panel data analysis, Correlated random effects estimator, Quality of life research, Unobserved heterogeneity

## Abstract

A plethora of research shows that income is an important factor in adult’s life satisfaction, but research ascertaining its importance for children’s life satisfaction is scant. Using a largescale nationally representative longitudinal survey with children aged 10–15, we estimate comprehensive life satisfaction models that account for heterogeneity in exogenous circumstances in children’s lives, focussing on family income and material deprivation. We find empirical support for the hypothesis that children are more satisfied with their lives, the more income their family has and the less material deprivation they experience throughout their teens. There are, however, differences across age groups with children aged 12–15 experiencing greater life satisfaction losses on account of lower family material wellbeing than younger children. Overall, income effects for older children are small but statistically significant when accounting for unobserved individual differences.

## Introduction

Following recommendations by world-leading economic advisors (e.g., Stiglitz et al., 2010), governments worldwide are looking to increase national wellbeing. Life satisfaction is widely accepted as an indicator of people’s overall experienced utility (van Praag et al., 2003) and has become a key performance indicator to this end. But what is important for life satisfaction, and how can government affect it? Life satisfaction research has ascertained several consistent relationships (e.g., Dolan et al., 2008): First, unemployment and a lower level of financial resources are associated with lower life satisfaction (e.g., Biswas-Diener, 2008; Howell & Howell, 2008; Lucas & Dyrenforth, 2006). Second, married people are more satisfied with life than never-married singles, divorcees (including those living in separation), and widowers (e.g., Shapiro & Keyes, 2008). People who belong to a religion are more satisfied (Lim & Putnam, 2010), and poor health is a significant factor in explaining lower life satisfaction (Diener et al., 1999). Intriguingly, satisfied people live longer and healthier lives (Diener & Chan, 2011). Thus, life satisfaction is a desirable outcome for persons at all stages of the lifecycle and for policymakers aiming to create policies that deliver the greatest happiness to all (Frijters et al., 2020; Kahneman & Sugden, 2005; Layard, 2005).

Nevertheless, the bulk of the life satisfaction research has examined correlates of life satisfaction during adulthood, treating age either as a mediator in determining the effects of socioeconomic factors (e.g., George et al., 1985), focusing on whether life satisfaction is U-shaped in age (e.g., Bartram, 2020; Blanchflower & Oswald, 2008), or on life satisfaction specifically in old age (e.g., Palmore, 1981). Although life satisfaction in childhood has also been examined since the mid-1990s (Antaramian et al., 2008), many aspects of life implicated in adult life satisfaction are not empirically explored among children. Ultimately, this risks that governments will not address issues that may pertain specifically to children.

One of the gaps in the empirical evidence regards the effect of family income on children’s life satisfaction. As a case in point, the comprehensive reviews of correlates of children’s life satisfaction by Proctor et al. (2009) and Antaramian et al. (2008) do not mention family income, while Holder (2012) reports findings from research with adults, not children. The scarcity of empirical evidence on this matter is predicated on the scarcity of largescale surveys that collect data on children’s life satisfaction and high-quality data on family income. Identifying income effects on life satisfaction is challenging, requiring large, powerful panel samples that can deal with heterogeneity in subjective evaluations and context. Ideally, studies also need to be able to account for unobservable differences between individuals that explain life satisfaction—such as personality (e.g., DeNeve & Cooper, 1998) and genes (e.g., Bartels, 2015)—as not accounting for them can bias the empirical results (Ferrer‐i‐Carbonell & Frijters, 2004).

Against this background, this research makes several important contributions. From the perspective of adult-focused life satisfaction research, this paper explores whether contemporaneous factors associated with adult life satisfaction are also associated with life satisfaction in children, focusing mainly on income, which is among the most-researched factors in adult life satisfaction (e.g., Ambrey & Fleming, 2014; Biswas-Diener, 2008; Caporale et al., 2009; Cheung & Lucas, 2015; Dolan et al., 2008; Frijters et al., 2004). From the perspective of the child life satisfaction literature, this research is among the first to report results on children’s life satisfaction using comprehensive life satisfaction models with fine-grained high-quality data on family income as regressors. Moreover, no study has exploited the full range of methodological advantages afforded by exploiting repeated information on life satisfaction from the same children. Factors such as personality and genes cannot or are generally not measured in surveys,^1^ but to the extent to which they are fixed over time, we can account for them by drawing on panel estimators (as we will do here). Last but not least, from the perspective of policymakers, given the current focus of many national governments on measuring population wellbeing (Frijters et al., 2020; Kahneman & Sugden, 2005; Layard, 2005), our results will provide robust empirical evidence on how children’s life satisfaction co-evolves with their family’s income. A positive association would suggest that re-distribution of income to families with children could play an important role in maximising population wellbeing, hence reinvigorate the focus on (cost) effective policy interventions to aid disadvantaged children.

## Life Satisfaction and Its Correlates

Life satisfaction is “a reflective appraisal, a judgment, of how well things are going, and have been going” Argyle (2001). Conceptually, life satisfaction is the cognitive component of subjective wellbeing, the other domains being positive affect (that is, the experience of emotions such as happiness and joy) and negative affect (that is, the experience of emotions such as sadness and loneliness) (Diener et al., 1999). A large number of instruments have been developed to assess affective and cognitive wellbeing in children (for reviews, see Bender, 1999; Pollard & Lee, 2003), and there is some empirical evidence that each component of subjective wellbeing can be measured accurately and consistently in children (Ravens-Sieberer et al., 2014).

Life satisfaction is also conceptualised as a catch-all measure of people’s perceived quality of life (or, in economic terms: utility). External (objective) factors play an important role when people appraise how well their life is going. This can be linked to the philosophical assumption that universal needs must be met for people to be happy. According to this view,^2^ people who find themselves in a ‘good situation’ for the fulfilment of needs are happy, while those who find themselves in a ‘bad situation’ are unhappy (Diener et al., 1999).

Adults consider seven aspects of their life when reporting their life satisfaction—their family-living context, health, financial situation, work-life, community and friends, personal values, and personal freedom (Layard, 2005). Many of these aspects have been shown to be important also for children’s evaluation of how well life is going, while others appear to be less well researched (in particular using largescale, nationally representative surveys with children). The family living context, being embedded in the (local) community, and friendships seem particularly salient to children’s life satisfaction. When British children (aged 11–15 years) were asked about one thing they would change to improve their life, they frequently mentioned interpersonal relationships with family and friends: children would like their parents to reunite, live with an absent parent or have fewer conflicts with siblings and friends (Scott & Chaudhary, 2003). Lack of activities in the home and neighbourhood and living too far from friends is also a recurring theme in the child-centred subjective wellbeing literature (Fattore et al., 2009). Although the neighbourhood context experienced in childhood impacts several structural inequalities in adulthood (for reviews, see, e.g., Ellen & Turner, 1997; Brooks-Gunn et al., 1993), its more immediate consequence for children’s life satisfaction has, to our knowledge, not been examined.

Research examining the role in children’s life satisfaction played by objective characteristics relating to the child’s school, health status, values and personal freedom is equally scant. There is, to our knowledge, no study investigating the link between child health and life satisfaction using objective measures of health.^3^ Regarding the school context, Gibbons and Silva (2011) explored whether past school performance predicted children’s life satisfaction in Britain at age 14 and found no statistically significant associations. In research with adults, the impact of personal values and personal freedom is typically operationalised using cross-national comparative studies exploiting information about minority ethnic group membership, immigration, or citizenship status (Layard, 2005). Some studies also exploit sex differences. Such cross-national multicultural analyses do not as yet exist for children.^4^ To the extent that mothers’ concepts of children’s areas of personal freedom are gendered (in favour of boys, see Nucci & Smetana, 1996; Yamada, 2004), sex may in itself mark differences in the level of personal freedom experienced by adolescents. In general, however, the evidence on sex differences in life satisfaction in the developmental stage of adolescence is inconclusive (Chen et al., 2020)^5^ and no studies have undertaken an in-depth analysis aimed at identifying the causes underpinning any identified sex gaps. Similarly, many studies report a negative association between life satisfaction and age among adolescents (see, for example, the meta-analysis by Chen et al., 2020). Still, the reasons for this association overall remain unexplored.

### Childhood Economic Circumstances and Childhood Outcomes

The focus of this research is on the impact of family income on children’s life satisfaction. Inequalities in structural outcomes for children growing up in poverty are a source of extensive research within and across countries and disciplinary boundaries. The bulk of the research has focused on the effects of family income on structural outcomes such as cognitive and behavioural development (see, e.g., Mayer, 1997; Blau, 1999) and concluded that children in lower-income households have worse outcomes than their more affluent counterparts: Income effects are more marked the lower the income, and outcomes are worse the more time has been spent in low-income households and the earlier in life the income shortfalls occurred (Cooper & Stewart, 2013, 2017). The effects of socioeconomic disadvantage manifest in structural outcomes assessed during both childhood and adulthood, suggesting that policies aimed at increasing child wellbeing may have a significant and lasting impact well into the future.

It is less clear how childhood economic circumstances impact life satisfaction. Recent British studies suggest that associations with life satisfaction assessed in adulthood may be small (Frijters et al., 2014; Layard et al., 2014; Stafford et al., 2016)^6^ and the comprehensive analysis of the relationship between children’s life satisfaction and family income is still in its infancy. Burton and Phipps (2008) used a cross-sectional, nationally representative sample of children aged 12–17 living in Canada. They showed that family income (measured in broad categories) is associated with low but not with high life satisfaction. Studies by Holder and Coleman (2008) and Sarriera et al. (2015) found a positive association between children’s life satisfaction and their subjective evaluation of their family’s income position.^7^ A further group of studies considered the neighbourhood or national financial situation instead of family income. Gadermann et al. (2016), using the median household income in the local neighbourhood as a proxy for the unobserved family income,^8^ found no statistically significant associations between income and children’s life satisfaction. In a comparative study, Levin et al. (2011) found that national income and income inequality predicted high life satisfaction among children. Gudmundsdóttir et al. (2016) compared children’s average life satisfaction levels before and after Iceland’s financial crisis. They found that children were, on average, more satisfied with life following the crisis despite lower household incomes.

Overall, the evidence on the relationship between family income and children’s life satisfaction, thus, is inconclusive.^9^ This is also a conclusion reached from various principally cross-sectional (sometimes time-series) analyses for Britain of bespoke or nationally representative surveys reported in the annual Good Childhood Reports (see, e.g., The Children’s Society 2019, p. 55). Strong associations between children’s life satisfaction and their family income tend to be found when both the outcome and dependent variables are subjective evaluations by the same child, and when the analysis does not take account of subjectivities in judgement, personality and genetic predispositions (or, in more technical terms: unobserved individual heterogeneity) which likely causes spurious correlations.

But there is also the issue that we may not expect children to know their family income, hence not identify any effects. A small number of studies, all using British data, have investigated the associations between children’s life satisfaction and family income and more concrete objective measures of material and social deprivation faced by family members.^10^ Knies, (2011), using preliminary data from the first wave of Understanding Society: the UK Household Longitudinal Study (UKHLS), showed no association between children’s life satisfaction and household income or the level of material deprivation faced by adults or children in the household. Later longitudinal analysis showed a small positive association for children aged 13–15 but not for those aged 10–12 (Knies, 2017). Markers of adult and child material deprivation, too, were not associated with life satisfaction. However, those children who had experienced more severe deprivation across their teens were significantly less happy with their lives than their less deprived counterparts. Knies (2017) ’s analysis uses the same analytical framework and estimation techniques we adopt in this paper. It also provides longitudinal evidence on what objective characteristics of living circumstances matter for children’s life satisfaction, focusing mainly on family income and other material wellbeing measures and investigating effect heterogeneity across children of different ages. However, the specific hypotheses regarding the age at which we might expect income effects to transpire are refined. The present analysis also uses the latest panel data (which observes more children enhancing the power to identify effects). As before, the analysis accounts for unobserved individual heterogeneity.

## Hypotheses

What would we expect to find? To the extent that a basic sustainable income is essential if individuals are to have access to resources needed to fulfil basic needs and participate in mainstream society, we may expect a positive relationship between income and life satisfaction for people at all life stages. For adults, this positive association has been well documented (e.g., Diener et al., 1993), including at levels of material wellbeing beyond the sheer necessary, but it is also true that income differences play only a minor role in explaining satisfaction gaps (Biswas-Diener, 2008). The relationship between income and life satisfaction may be even weaker for children than it is for adults. Unlike adults, children may not view the family income as a sign of their success (Burton & Phipps, 2010).

Moreover, there is empirical evidence that parents shield their children from financial hardship by spending on their children rather than themselves (Lister, 1996). This shielding may mislead the children in assessing their family’s financial situation and consequently blur the association between family income and life satisfaction. Against this background, we hypothesise that household income is not associated with children’s life satisfaction because children do not know how much income the family has or how it has changed over time (*H1*).

When lack of income means that families cannot afford to engage in activities or consume things that others have, in other words, when lack of income impacts more concrete material and social factors (Cummins, 2000), this may not go unnoticed by children and, in turn, affect their quality of life. The associated discontent may be most marked if children are excluded from activities and goods enjoyed by others in their age group. In the empirical analysis, we will investigate this hypothesis by focusing on the levels of material deprivation experienced by the adults and children in the household separately. We hypothesise that material deprivation is visible to children of all ages, and the higher the deprivation, the lower their satisfaction (*H2*). Secondly, the material deprivation experienced by adults in the household will affect children’s life satisfaction less than the personally experienced child material deprivation (*H3*). Thirdly, we hypothesise that material deprivation hurts more when the adults (respectively, children) have to go without activities and goods that a greater share of the population has (*H4*).

Our final set of hypotheses (*H5* to *H7*) relates to interactions between the child’s age and the relationship between income, material wellbeing and life satisfaction. As children get older, the family income and its association with quality of life may become more relevant. Not only do children’s consumption needs grow (Hirsch et al., 2012), older children may understand the value of money better, in particular when they start contributing to meeting their income needs by doing part-time work or get rewarded for completing chores.^11^ As children gain agency, they can also be assumed to be less likely to evaluate life as good if it objectively is not: As opportunities for changing one’s situation increase, people are less likely to accept bad situations without resentment (Ipsen, 1975). In line with cognitive development theories, we hypothesise that children who have entered the phase of early adolescence (that is, when they are aged 12 or above, see University of Rochester Medical Center 2020; Piaget & Inhelder, 1966) have the cognitive ability to link the family income to how much pocket money and access to activities and facilities they have and what this means for their wellbeing. Specifically, we expect a positive association between household income and life satisfaction among children aged 12–15 and negative associations with all other material deprivation measures (*H5*). In early adolescence, we would expect children to care even more about their personal experience of deprivation than about the deprivation faced by adult household members. In other words, we expect the differences in effect sizes between adult and child material deprivation to be more marked in this group than across all children (*H6*). Last but not least, it is a well-established finding in the happiness research that people care about their material position relative to similar others (e.g., Knives et al., 2008; Clark et al., 2008). As older children are influenced more by their peer group than younger children, we expect the effect of material deprivation on life satisfaction to be more marked for older children than for younger children, particularly if children go without items that a larger share of their peers have (*H7*).

## Data and Methods

This research draws on data from the first nine waves of Understanding Society, the UK Household Longitudinal Study (UKHLS), a multi-focus multi-topic longitudinal household panel study that started in 2009 with a nationally representative, stratified and clustered sample of 40,000 households (University of Essex, 2019). Fieldwork takes place over 24 months, with a random sample of households issued for interview each month. Within each household, all those aged 10 and above are eligible for annual interviews. Children aged 10–15 are invited to complete a youth self-completion interview. By wave nine, 13,345 children (that is, ~ 70 per cent of eligible children in productive households) had followed the request, providing a total of 35,225 interviews. For more detailed information about the study, see (Knives 2018).

Our outcome variable, life satisfaction, is collected annually in the youth self-completion questionnaire based on a 7-point scale running from 1 [very satisfied] to 7 [very dissatisfied], where more or less smiling faces represent categories. Children are asked to tick the box, which best describes how they feel about their life as a whole. We reverse-code this, so higher values represent greater satisfaction with life, as is customary in life satisfaction research.

Household income is a measure of net monthly household income, deflated using the modified OECD equivalence scale, and adjusted for inflation using monthly consumer price indices (100 = 2015). Households with less than £1 income and those in the 1st or 99th percentile of the household income distribution are excluded. Household income is computed by drawing on income from employment and other sources reported by adults in the household and routinely provided with the data. For further detail, see Fisher et al. (2019).

We use two material deprivation indicators to throw some more light on whether and how a lack of financial resources affects children’s satisfaction with life. The head of the household is asked to report whether all adults (respectively, all children aged 0–15 years) in the household have a range of goods and activities considered by a majority of the population as necessities for adults (respectively, children) to participate in mainstream society. To generate the adult and child material deprivation indices, each unaffordable item is assigned a value of 1 (else 0), then summed and divided over the total number of items. For "weighted" indices, we multiplied each lacked item by the proportion of the population that has the item, summed all weighted items, and divided over the total number of items. The idea behind weighting is that not having the good or activity may hurt more the more prevalent doing the activity/having the item is. Weighted indices can range from 0 to 1, with 1 representing a household lacking all items that everybody else has. The deprivation measures are only collected in the first and second waves of the UKHLS and then bi-annually in even-numbered waves. To fill the data gap in the odd-numbered waves, we use the average deprivation score observed in the child’s households as impute.^12^

Basic socio-demographic characteristics (age, sex, and ethnicity) are included alongside two markers of the family composition (family type and the number of children in the household). To absorb any effect of schools on children’s wellbeing, we include a proxy for whether or not the interview took place during school holidays.^13^ The argument is that if the net effect of going to school is positive, the effect of holidays will be negative and vice versa. Children’s community contexts are captured by the ACORN classification of neighbourhoods (CACI, 2013),^14^ and by whether a child lived in a different neighbourhood^15^ in the previous wave (yes = 1, else 0).

Given the complex study design, all models include (a) the calendar year to capture general trends in life satisfaction, (b) a dummy for whether the interview took place in an even or uneven wave to remove any biases resulting from the questionnaire in uneven waves focusing on somewhat more negative aspects of children’s life (1 if uneven wave, else 0), and (c) and a dummy to allow for the effect of participating for the first time in the interview (1 if first interview, else 0), which has been shown to exist in life satisfaction reports (Frick et al., 2006). To be included in the analysis, children had to have complete data on all relevant variables. Table 1 reports descriptive statistics of indicators used in the analysis.

**Table 1:** Sample description

	Mean	S.D	Min	Max
Life satisfaction	5.85	1.17	1	7
Household income	1,383	736	150	6,603
Adult Material Deprivation Index	0.29	0.30	0	1
Adult Material Deprivation Index—weighted	0.16	0.18	0	1
Child Material Deprivation Index	0.09	0.14	0	1
Child Material Deprivation Index—weighted	0.06	0.10	0	1
Age	12.61	1.68	10	15
Female	0.50	0.50	0	1
British/Irish white	0.70	0.46	0	1
Family type				
Both biological parents	0.67	0.47	0	1
Step family	0.13	0.33	0	1
Single parent family	0.21	0.41	0	1
Number of children in household	2.15	1.08	1	10
Interviewed during term time	0.77	0.42	0	1
Neighbourhood type				
Affluent Achievers	0.23	0.42	0	1
Rising Prosperity	0.06	0.23	0	1
Comfortable Communities	0.27	0.44	0	1
Financially Stretched	0.23	0.42	0	1
Urban Adversity	0.21	0.41	0	1
Moved to different neighbourhood	0.03	0.17	0	1
Number of observations	24,375			

*Source*: Understanding Society (2019), Wave 1–9, 2009–2018, linked with neighbourhood and school holiday indicators for England

### Empirical Strategy

To analyse the association between life satisfaction and family income, we will first examine bivariate relationships. We will then estimate life satisfaction models that control for objective heterogeneity in those aspects of children’s lives linked to their life satisfaction. In statistical terms, the model can be written as

$$y_{it} = \beta^{\prime}X_{it} + \gamma^{\prime}\overline{X}_{i} + \epsilon_{it} + \omega_{i}$$

with $i$ denoting individuals and $t$ denoting time. The outcome variable $y_{it}$ denotes life satisfaction for child $i$ at time $t$. X is a vector of exogenous characteristics that are held to influence the life satisfaction of child $i$ at time *t*. $\overline{X}$ is a vector of the means of the exogenous characteristics for child $i$ over the observation period 1,.., T. There is an unknown component comprised of a random error term (${\upvarepsilon }_{it}$) and a person fixed effect (${\upomega }_{i}$). The model assumptions are that ${\upomega }_{i}$ is normally distributed with mean zero and standard deviation $\sigma_{w}$ and uncorrelated with $\overline{X}_{i}$ or $X_{it}$. The so-called correlated Random Effects (RE) model is a middle ground between the so-called Fixed Effects (FE) model that is flawed in a case such as ours when we only have a small number of observation points per respondent and the (uncorrelated) RE model, which is flawed by the assumption that the error terms are uncorrelated with the observed regressors (Mundlak, 1978). For not time-varying characteristics (such as sex and ethnicity), the correlated RE model produces the same coefficients as the RE model, in which coefficients express how much the outcome differs at different levels of x. For characteristics that change over time (such as household income), the correlated RE model produces the same coefficients as the FE model, in which coefficients express how much a unit change in x is associated with a unit change in the outcome. For all time-varying characteristics, the correlated RE model reports both types of coefficients; that is, the overall effect observed in the cross-sectional models is disentangled into the longitudinal effect exploiting "within"-variation and the cross-sectional effect exploiting "between"-variation, respectively. In a strictly balanced panel with t = 2, the two coefficients add up to the coefficient from the (uncorrelated) RE model.

Our outcome variable life satisfaction is measured on an ordinal scale; however, we treat it as continuous for parsimony. All standard errors are adjusted to account for heteroscedasticity and within-person serial correlation.

Analysis proceeds in two stages. To set the scene, we provide population estimates about children’s life satisfaction and their living circumstances over the observation period. We then estimate multivariate longitudinal panel models to test empirically the specific hypotheses set out above. To test the first four hypotheses, we will compare the results from models that include each of the five material wellbeing indicators separately. To test the age-related hypotheses (*H5-H7*), we will interact each material wellbeing indicator with whether the child is aged 12–15. In these specifications, the main effect of income (respectively, deprivation) captures the effect on younger children’s life satisfaction (that is, when the age 12–15 dummy assumes a value of 1). The interacted effect captures the effect on older children.

## Results

### Change Over Time in Key Indicators

**Fig. 1:**
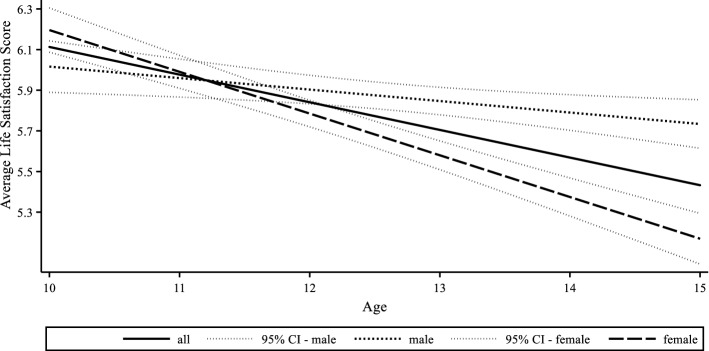
Life satisfaction trajectory for children aged 10–15

Our identification strategy relies on observing changes in children’s life satisfaction and living circumstances. Figure 1 shows the average life satisfaction for children aged 10–15 who participated in six consecutive interviews during the observation period (that is, 2009–2018). We can see that life satisfaction at age 10 reduces from an average of just over six points to 5.6 points when children are aged 15. The satisfaction loss throughout the early teens is statistically significant, with children being more satisfied with life on average when they are 10 or 11 rather than 12–15 years old. A further breakdown by sex shows a steeper fall in life satisfaction for girls than for boys, on average.

Table 2 presents basic information about stability and change in children’s material resources. Children experienced a considerable degree of change in household income over the observation period, with 74.2 (third decile) to 41.2 (top decile) of them moving from one decile of the income distribution to another decile within a year. By contrast, movement into and out of high material deprivation—defined here as lacking more than 25 per cent of the listed items and dubbed 'deprived'—is relatively low. Almost every other child lives in a household (44.6 per cent; weighted index: 27.7 per cent) in which the adults are deprived, and the vast majority of them (81.2 per cent; weighted index: 68.2 per cent) are still faced with deprivation in the following year. The proportion of children facing high levels of child deprivation is considerably lower: 12.4 per cent of children lack more than 25 per cent of the items, and 46.2 per cent of them no longer experience this high level of deprivation in the following year. A smaller proportion of children experience high deprivation when individual items are weighted (5.6 per cent), and a greater share of them are no longer experiencing this high level of deprivation the following year (57.6 per cent). Whilst high rates of mobility out of high deprivation suggest that children’s quality of life might have improved, it is also true that 14.4 per cent (weighted index: 8.9 per cent) of children start experiencing adult and 5.2 per cent (weighted index: 2.8 per cent) child material deprivation in the family.

**Table 2:** Stability and change in children’s life circumstances 2009–2018

Child characteristic in year t	Pooled cross-section	Transitions from year t to t + 1	
		x_t_ = x_t+1_	x_t_ ≠ x_t+1_
Life satisfaction			
Very dissatisfied (1)	0.7	10.8	89.2
2	1.1	9.8	90.2
3	2.5	15.1	84.9
Neither dissatisfied nor satisfied (4)	7.8	22.5	77.5
5	18.1	34.3	65.7
6	36	47.6	52.4
Very satisfied (7)	33.8	53.4	46.6
Household income			
Bottom decile	12.9	47.8	52.2
2nd decile	12.6	29.9	70.1
3rd decile	12	25.8	74.2
4th decile	11.8	27	73
5th decile	11.2	27.3	72.7
6th decile	10.1	28.6	71.4
7th decile	9.1	30.4	69.6
8th decile	7.9	33.8	66.2
9th decile	6.6	39.8	60.2
Top decile	5.7	58.8	41.2
Adult Material Deprivation Index > 25%			
Not deprived	55.4	85.6	14.4
Deprived	44.6	81.2	18.8
Adult Material Deprivation Index—weighted— > 25%			
Not deprived	72.3	91.1	8.9
Deprived	27.7	68.2	31.8
Child Material Deprivation Index > 25%			
Not deprived	87.6	94.8	5.2
Deprived	12.4	53.8	46.2
Child Material Deprivation Index—weighted— > 25%			
Not deprived	94.4	97.2	2.8
Deprived	5.6	42.4	57.6

*Source*: Understanding Society (2019), Wave 1–9, 2009–2018, linked with neighbourhood and school holiday indicators for England

### Multivariate regression analysis

Table 3 shows the results of multivariate regressions on children’s life satisfaction. To demonstrate the mechanics behind the models, we present the results of the correlated RE model alongside those from the more familiar OLS, RE and FE models. We can see that for not time-varying characteristics, the correlated RE model produces the same coefficient as the RE model (e.g., β_[female]_ = − 0.12). For characteristics that change over time, it has the same coefficient as the FE model (e.g., β_[household income]_ = − 0.02). For readability, we do not report the cross-sectional ‘between’ effect for time-varying characteristics here.

**Table 3:** Multivariate longitudinal regressions of exogenous life circumstances on children’s life satisfaction

	Pooled OLS			Random effects			Fixed effects			Correlated random effects		
	β-coef.	S.E	*p* value	β-coef.	S.E	*p* value	β-coef.	S.E	*p* value	β-coef.	S.E	*p* value
Household income (log)	0.03	0.023	0.228	0.02	0.020	0.453	− 0.02	0.030	0.488	− 0.02	0.030	0.488
Age (Ref: 10 years old)												
11 years old	0.04	0.026	0.102	0.02	0.025	0.335	0.05	0.028	0.097	0.05	0.028	0.097
12 years old	− 0.06	0.028	0.039	− 0.08	0.027	0.005	0.03	0.035	0.418	0.03	0.035	0.419
13 years old	− 0.16	0.030	0.000	− 0.19	0.028	0.000	− 0.01	0.044	0.840	− 0.01	0.044	0.841
14 years old	− 0.25	0.030	0.000	− 0.28	0.029	0.000	− 0.02	0.054	0.661	− 0.02	0.054	0.661
15 years old	− 0.31	0.032	0.000	− 0.35	0.030	0.000	0.00	0.066	0.982	0.00	0.066	0.982
Female	− 0.12	0.021	0.000	− 0.12	0.020	0.000				− 0.12	0.020	0.000
British/Irish White	− 0.04	0.025	0.093	− 0.05	0.024	0.055				− 0.05	0.024	0.038
Family type (Ref: biol. parents)												
Step family	− 0.23	0.033	0.000	− 0.23	0.032	0.000	− 0.23	0.080	0.003	− 0.23	0.080	0.003
Single parent family	− 0.20	0.029	0.000	− 0.22	0.027	0.000	− 0.25	0.140	0.077	− 0.25	0.140	0.077
Number of children in household	0.02	0.023	0.292	0.03	0.021	0.184	0.01	0.044	0.759	0.01	0.044	0.759
No school holidays	0.06	0.019	0.001	0.06	0.017	0.000	0.05	0.019	0.009	0.05	0.019	0.009
Neighbourhood (Ref: Comfortable Communities)												
Affluent Achievers	0.08	0.029	0.007	0.06	0.027	0.016	0.00	0.064	0.972	0.00	0.064	0.973
Rising Prosperity	0.02	0.042	0.640	0.01	0.041	0.761	0.00	0.110	0.975	0.00	0.110	0.975
Financially Stretched	− 0.04	0.030	0.181	− 0.04	0.029	0.195	0.05	0.079	0.550	0.05	0.079	0.550
Urban Adversity	− 0.02	0.032	0.436	0.00	0.030	0.926	0.32	0.096	0.001	0.32	0.096	0.001
Moved to different neighbourhood	− 0.07	0.048	0.123	− 0.06	0.043	0.182	− 0.04	0.047	0.394	− 0.04	0.047	0.394
Design & time trend	Yes			Yes			Yes			Yes		
Mean values of context variables	No			No			No			Yes		
Constant	6.13	0.172	0.000	6.29	0.153	0.000	7.47	0.284	0.000	6.00	0.208	0.000
Number of observations	24,370			24,370			24,370			24,370		
R-squared (overall)	0.03			0.03			0.01			0.03		
R-squared (within)				0.04			0.04			0.04		
R-squared (between)				0.03						0.03		

*Source*: Understanding Society (2019), Wave 1–9, 2009–2018, linked with neighbourhood and school holiday indicators for England

The results from the correlated RE model suggest that, compared to living with both biological parents, children are no less satisfied with life when they live in a step-family [*p* = 0.077]. Still, they are less satisfied when they live in a single-parent family [*p* = 0.003]. Moreover, females are less satisfied with their life, as are children who identify as British/Irish White. Life satisfaction is higher among 10- and 11-year-olds and then reduces year on year. Being at home outside term time^16^ is associated with satisfaction losses, *ceteris paribus*, and children who live in neighbourhoods classified as ‘Urban Adversity’ are more satisfied with their life than children who live in ‘Comfortable Communities’ (the reference category). Having moved to a new neighbourhood in the last 12 months does not appear to impact children’s life satisfaction (but this is a rare event; only 2.9 per cent of our sample experienced a move; 10.7 per cent of them moved again in the following year, see “Appendix”).

All effects mentioned above are remarkably stable across the four model specifications—indicating that they do not suffer from stark biases due to unobserved heterogeneity—and we will now focus on the main effect of interest: that of family income. Here, the results suggest that there is a positive association between children’s life satisfaction and income in the cross-sectional pooled OLS and RE models but, in line with our first hypothesis (*H1*), the effect is not statistically significant when we account for unobserved differences between individuals: In the FE and correlated RE models, the (longitudinal ‘within’) effects do not reach statistical significance.

### Results of Hypotheses Tests

Further tests about the effects of income and other markers of material wellbeing on children’s life satisfaction are reported in Table 4. For convenience, we only report the longitudinal ‘within’ effects (left panel) and cross-sectional ‘between’ effects (right panel) for income and material wellbeing from the most efficient correlated RE model.

**Table 4:** Multivariate correlated random effects regressions of children’s life satisfaction on material wellbeing

Material wellbeing indicator used in model		*x*			$\overline{x}$		
		β-coef.	S.E	*p* value	β-coef.	S.E	*p* value
I	Household income (log)	− 0.02	0.03	0.472	0.04	0.05	0.392
II	Adult Material Deprivation	− 0.06	0.05	0.249	− 0.23	0.09	0.028
III	Child Material Deprivation	− 0.08	0.10	0.403	− 0.51	0.18	0.021
IV	Adult Material Deprivation—weighted	− 0.10	0.08	0.241	− 0.36	0.14	0.029
V	Child Material Deprivation—weighted	− 0.12	0.13	0.370	− 0.69	0.25	0.024
VI	Child Material Deprivation—items—weighted						
	Holiday away from home	− 0.07	0.05	0.147	− 0.09	0.07	0.159
	Own bedroom	0.13	0.07	0.077	− 0.06	0.10	0.595
	Celebrations at special occasions	0.01	0.06	0.927	− 0.05	0.09	0.564
	A hobby/ leisure activity	− 0.05	0.09	0.573	− 0.06	0.13	0.624
	Have friends around for tea	− 0.11	0.07	0.109	− 0.09	0.11	0.433
	Go on school trips	0.08	0.08	0.311	− 0.39	0.13	0.002
	Leisure item such as a bicycle	− 0.06	0.10	0.534	− 0.09	0.17	0.593
	Effects for children aged 12–15						
VII	Household income (log)	0.11	0.03	0.001	0.04	0.07	0.595
VIII	Adult Material Deprivation	− 0.11	0.05	0.040	− 0.06	0.12	0.640
IX	Child Material Deprivation	.28	0.12	0.017	− 0.10	0.26	0.690
X	Adult Material Deprivation—weighted	− 0.19	0.09	0.038	− 0.02	0.19	0.906
XI	Child Material Deprivation—weighted	− 0.38	0.16	0.019	− 0.16	0.36	0.654
	Effects for children aged 10–11						
VII	Household income (log)	− 0.13	0.04	0.000	0.13	0.06	0.040
VIII	Adult Material Deprivation	0.03	0.06	0.594	− 0.31	0.10	0.003
IX	Child Material Deprivation	0.12	0.13	0.345	− 0.54	0.23	0.017
X	Adult Material Deprivation—weighted	0.12	0.10	0.190	− 0.61	0.17	0.000
XI	Child Material Deprivation—weighted	0.15	0.17	0.402	− 0.72	0.31	0.020

All models additionally include controls for age (continuous), sex, British/Irish white ethnicity, family type, number of children in household, whether the interview took place during the school holidays, neighbourhood type, mover status, survey design and year. Models VII to XI additionally include the two interacted variables (i.e., a dummy for aged 12–15 and the respective income or material deprivation indicator). The number of person-year observations in each model is N = 24,375

*Source*: Understanding Society (2019), Wave 1–9, 2009–2018, linked with neighbourhood and school holiday indicators for England

Turning to our hypotheses regarding material deprivation, the results indicate weak support for the hypothesis that material deprivation is associated with children’s life satisfaction (*H2*). The longitudinal and cross-sectional effects on the adult and the child material deprivation indices (see models II and III, respectively) are negative, albeit only the cross-sectional effects are statistically significant. This means that children who experienced, on average higher levels of deprivation throughout their teens are less satisfied with their lives than children who have experienced lower levels of deprivation. In terms of effect sizes, the coefficients on child material deprivation are almost twice as large as the coefficients on adult material deprivation, which lends some support to the hypothesis that the children’s own material situation matters more for their life satisfaction than that of adults in their household (*H3*). However, the differences in the cross-sectional effects are not statistically significant, and we do not observe any differences in the size of the longitudinal effects.

Associations are stronger when we use the weighted indices (see models IV and V) than when we use unweighted indices (see models II and III). This is in line with the hypothesis that life satisfaction gaps are more marked if children go without the things that are more readily available to other families than if they go without things that more other families also do not have access to (*H4*). Again, we do not find that these differences are statistically significant at the 5 per cent level. In an additional specification (see model VI), we tested whether any items included in the child material deprivation index stood out as a driver of dissatisfaction. We found that only one of the seven items were related to children’s life satisfaction; namely: not participating in school trips was associated with a 0.36 reduction in life satisfaction [*p* = 0.002].

To test our final set of hypotheses—that is, that the family’s material situation will affect older children more—we interacted the five material wellbeing indicators with being aged 12–15. In these specifications, the main effect of income (respectively, deprivation) captures the effect on younger children’s life satisfaction (that is, when the age 12–15 dummy assumes a value of 1). The interacted effect captures the effect on older children. The results for older children are reported in the middle section, those for younger children at the bottom of Table 4.

In line with our hypothesis, we find that the longitudinal associations between life satisfaction and income (see model VII) and material deprivation (see models VIII and IX, respectively) are statistically significant for the group of children aged 12–15 *(H5).* Differences between the weighted and unweighted adult and child deprivation coefficients suggest that the children’s personal experience of deprivation is more important than the adult household members’ experience of deprivation but are not statistically significant *(H6).* Last but not least, we find no clear empirical support for the hypothesis that the effect sizes are larger for older children than for younger children (*H7*): None of the relevant longitudinal material deprivation coefficients is statistically significant for younger children (see models VIII to XI, bottom part of Table 4). However, identifying these specific effects is very challenging as it relies entirely on observed changes in children aged 10 in year t and 11 in year t + 1 (that is, a very small subset of the analysis sample). The more easily identifiable cross-sectional effects follow the expected pattern in the younger age group.

## Discussion

This analysis has examined whether family income matters for children’s life satisfaction. As previous research has shown, identification of income effects on life satisfaction is cumbersome. Not only is there a great deal of heterogeneity in observed individual characteristics that correlate with income and explain life satisfaction—such as the family living context (e.g., Vignoli et al., 2014) and the work context (e.g., Brereton et al., 2008; Clark, 2010), but there is also relevant heterogeneity in unobserved characteristics—such as personality (e.g., DeNeve & Cooper, 1998) and genes (e.g., Bartels, 2015). To minimise omitted variable bias, in the life satisfaction research with adults, it is common practice to include markers for all aspects of life that adults consider when evaluating how well their life is going and, whenever possible, to estimate panel models that account for unobserved not time-varying characteristics (such as so-called Fixed Effects models, see, e.g., Ferrer‐i‐Carbonell and Frijters 2004). In the life satisfaction research with children, such comprehensive analyses are scant, with the bulk of the quantitative research reporting bivariate correlations.

Against this background, the current research presented a life satisfaction model that can show whether family income matters for the life satisfaction of children aged 10–15 years living in England, controlling comprehensively for observed and unobserved individual characteristics, disentangling the longitudinal effect and cross-sectional associations. Longitudinally (‘within’), we compare the same child’s life satisfaction over time as their family’s income changes, and the coefficient tells us how, on average, a child’s life satisfaction changes as the family income changes. Cross-sectionally (‘between’), we compare different children’s life satisfaction at given person-averaged levels of family income with each other. For policymaking, the longitudinal effect is more informative as this indicates how much more satisfied children would be if their own families received more income. The cross-sectional effects are more difficult to interpret (and often not reported by economists). For example, the person-average of child material deprivation tells us how much deprivation a child has experienced throughout their teens on average. This may proxy for the parents’ (unobserved) ability to spend the family money in ways that promote their children’s wellbeing, but it may also proxy a host of other things.

The baseline results (for children across the whole age range) suggest a strong and sizeable positive association between family income and children’s life satisfaction in the cross-sectional models. However, the effect is wiped out in the more sophisticated longitudinal models, which account for unobserved heterogeneity. The attenuation in the size of the income effects when controlling for unobserved heterogeneity is consistent with the life satisfaction research with adults (Ferrer-i-Carbonell & Frijters, 2004). That there is no empirical support for the hypothesis that income matters for children’s life satisfaction, too, is not surprising. Indeed, some authors have suggested that children may not know their family income (Knies, 2017) or not care so much about it (Burton & Phipps, 2010), prompting us to test whether the more visible material deprivation matters for children’s life satisfaction and more specifically, whether deprivation experienced by adults in the household, matters less than the deprivation experienced by the children themselves.

The strong and statistically significant negative associations with the average levels of deprivation experienced by the children throughout their early teens (cross-sectional effects when unobserved heterogeneity is accounted for) suggest that the deprivation of adults and children in the household is negatively correlated with children’s life satisfaction. While children do not appear to be more affected by their own deprivation compared to that of adults in their household, both types of deprivation are associated with life satisfaction losses, and comparison of estimates from models that used weighted deprivation indices with those from unweighted indices suggests that not having items that more others have hurts children more. This could indicate that children care about their relative standing, like adults (Diener et al., 1993).

We then drew on cognitive development theory (Piaget & Inhelder, 1966), arguing that children from the age of twelve may be able to know their family income given their greater consumption needs (Hirsch et al., 2012) and greater agency (Ipsen, 1975) may have given them a more concrete experience of the value of money. Previous studies (Knies, 2017) had chosen more arbitrary age-thresholds or included yet older children (e.g., Burton & Phipps, 2008) who in some studies (such as the one exploited here) would be considered adults. The empirical results offer some support for the hypothesis that older children are more able than younger children to perceive the value of money and to experience discontent when they have less of it, showing statistically significant longitudinal effects of income on 12–15 years-old children’s life satisfaction and no effects on 10–11 years-old children’s life satisfaction.

## Limitations

While the current study uses detailed, high-quality measures of household income where previous studies have used coarse measures such as categories of household income (Burton & Phipps, 2008), proxies for household income (Gadermann et al., 2016; Levin et al., 2011) or, more problematically, the child’s subjective evaluation of their family’s financial standing (e.g.,Main, 2019; Rees et al., 2011), we, too, were limited by the design of the survey. In particular, material deprivation is not measured in every wave of the survey. We addressed this missing data problem by using the child’s household’s average deprivation observed across all study waves as imputes.

The survey asks the responsible adult about the deprivation experienced by all children in the household (that is, instead of asking the parent about each child separately or by asking the children themselves), hence misses any variation in the extent to which children vary in their access to financial resources within and outside the household (e.g., some children may have a more affluent absent parent than others) and in their tastes and needs (e.g., some children may want items that the parent thinks the child does not want). This is, of course, true for all adults-reported measures. On the positive side, to the extent that these unobserved differences in the children’s preferences and their parents’ (potentially inaccurate) evaluations are stable over time, our panel estimators take them into account.

Nonetheless, it is challenging to identify longitudinal effects in short runs of panel data. Many of the children in our sample are only observed twice in five years (and not necessarily in consecutive waves). Overall, child material deprivation is also a rare phenomenon, possibly because parents shield their children from material deprivation (Lister, 1996). That this may indeed be the case is evidenced by the much higher levels of and persistence in adult deprivation compared to child deprivation: Only a small number of children are affected by child material deprivation at any point in time (the mean level in our sample is 0.12).

## Conclusions and outlook

Notwithstanding the limitations mentioned above, the data is a remarkable resource that opens up many exciting routes for longitudinal research into what matters for children’s life satisfaction. Future analysis may explore, for example, the dynamics of household income and its effects on child wellbeing. For dynamic analysis, it will be helpful also to explore whether there are threshold effects, that is, particular points in the income distribution at which child wellbeing is impacted the most. The Understanding Society study also collects additional information that would help further explore the notion that children value income more when they gain more agency and a better understanding of the value of money. This includes information about pocket money and whether children are doing any part-time jobs and an assessment of children’s cognitive ability for the first time in the tenth round of interviews.

Including some of the less frequently explored aspects of life that may matter for child wellbeing also uncovered some promising new routes for a future sociological inquiry. Using the survey interview dates and information on public holidays to compute whether the children were interviewed during term time showed that children are unhappier during school holidays, *ceteris paribus*. This could signify the effects of friends not being around, regular activities such as playing team sports not taking place, and reduced opportunities to get about (e.g., bus services are adjusted to lower demand); aspects which children have mentioned inhibit their quality of life, in qualitative research. A focus on holidays and the effects on wellbeing will be able to throw more light on this. It will be interesting to see, in due course, how the COVID-19 and associated lockdown affected children’s wellbeing; the Understanding Society study, which conducts interviews all year round, will be a superb resource to do so. Second, through the linkage of annual neighbourhood indicators, we explored whether neighbourhood contexts play a role in children’s life satisfaction. Somewhat surprisingly, we found that children are happier in neighbourhoods where social and economic problems culminate, *ceteris paribus*. It may be that increased investments into such areas (e.g., as part of the New Deal for Communities which were in place from 1999 to 2011) impacted children’s quality of life positively or, more generally, that urban areas offer more activities, often provided for free, than more comfortable communities that are otherwise privileged. Again, it is beyond the scope of this analysis to explore this further.

Overall, the results suggest several groups that governments who are looking to increase national wellbeing may target mainly: Children who are British/Irish White, female, and those in single-parent households. While governments may focus on redistributing income to families with older children to increase children’s subjective wellbeing, overall, the expected effect will be small (although we would expect there to be the small additional effect of having more income on the life satisfaction of adults in these households, too). Greater wellbeing impacts may be expected from providing children with more opportunities to do things, particularly during the holidays, and if the family cannot afford to spend time away from home.

## Appendix

Stability and change in children’s life circumstances 2009–2018—additional characteristics included in the multivariate models.

**Table:**

Child characteristic in year t	Pooled cross-section	Transitions from year t to t + 1	
		x_t_ = x_t+1_	x_t_ ≠ x_t+1_
Family type			
Both biological parents	66.6	98.3	1.7
Step family	12.7	94.8	5.2
Single parent	20.7	96.8	3.2
Interview period			
Holidays	22.7	38.8	61.2
Term time	77.3	81.5	18.5
ACORN type			
Affluent Achievers	23.2	97.3	2.7
Rising Prosperity	5.8	93.2	6.8
Comfortable Communities	26.8	95.9	4.1
Financially Stretched	22.7	94.8	0.0
Urban Adversity	21.4	96.5	3.5
Neighbourhood mobility			
Same neighbourhood last year	97.1	95.9	4.1
Different neighbourhood last year	2.9	10.7	89.3
Number of observations	24,375	15,475	

*Source*: Understanding Society (2019), Wave 1–9, 2009–2018, linked with neighbourhood and school holiday indicators for England

## Footnotes

1 Even if personality was, including it as a regressor would create endogeneity concerns.

2 For alternative philosophical theories, see Brief et al. (1993).

3 Some nationally representative surveys include measures of children’s satisfaction with their health. In a cross-national study of children’s satisfaction with various aspects of their life, Bradshaw and Richardson (2009) observed that children in the UK are unhappier with their health than children in other countries. Still, the survey did not include objective markers of children’s health that would allow us to model the impact of children’s health on life satisfaction using comprehensive life satisfaction models comparable to those estimated for adults.

4 Multinational-multicultural studies such as the Children of Immigrants Longitudinal Survey in Four European Countries (CILS4EU) could provide some insights for immigrant children but do not include children from majority (non-immigrant) backgrounds. Cross-national longitudinal analyses of children’s life satisfaction will be facilitated, in due course, by the new European cohort study Growing Up in Digital Europe (GUIDE).

5 This review notes that European studies show more pronounced sex gaps in life satisfaction than studies from other world regions (male adolescents tend to be more satisfied with life than female adolescents). Note that the screening used in this review will have missed studies that do not mention gender or sex in the title and that it includes studies that examined satisfaction with different domains of life.

6 Cf. Gariepy et al. (2017), who focus on the association between low income and a broader measure of subjective wellbeing, assessed at one point in time in an American sample of adolescents aged 12–19. The authors found a negative association, particularly if low income was experienced when aged 0–2.

7 Note that both the outcome and dependent variable are subjective evaluations by the same child. This means both are prone to the same subjectivities in judgement (Veenhoven 1996), likely causing a spurious correlation.

8 Using neighbourhood income as a proxy for household income builds on the assumption that neighbours are homogenous concerning their income. This may well not be the case. Several studies under the umbrella of testing the relative income hypothesis have shown that neighbourhood income has an independent effect on life satisfaction in models that also control for household income (e.g., Knies, 2012; Clark et al., 2009).

9 The same applies to the research which examines income effects on other subjective wellbeing outcomes in children (Gariepy et al., 2017; Main 2019; Rees et al., 2011).

10 For results reporting associations between the child’s subjective evaluation of their deprivation status and life satisfaction, see, e. g., The Children’s Society (2019), chapter 2.

11 Part-time work is legal from the age of 13 in the UK. A survey of 6–17-year-old’s parents suggests that the prevalence of pocket money is highest at the age of 12 (average monthly amount received: £17. 61); 54 per cent of parents give pocket money ‘for doing chores’, see https://www.ybs.co.uk/savings/passing-financial-torch/index.html. Date accessed: 19.01.2021.

12 The substantive results on material deprivation are not affected by the imputation: the bulk of the effects and differences in effect sizes are statistically insignificant whether we impute or not. It is beyond the scope of this analysis to develop a more sophisticated multiple imputation model for material deprivation.

13 Information on school holidays was sourced from a local government in East Anglia. There may be some misclassification of children having holidays because their school follows a slightly different schedule; the school flexibly determines five holidays a year.

14 We use the 6-category version but dropped the few cases living at addresses classified as commercial addresses as the address in the survey database may not be the family’s home address.

15 This is operationalised by whether or not the child’s address is in the same Lower Super Output Area (LSOA). LSOA are a census output unit of approximately 1,500 residents and are widely considered a good approximation of residential neighbourhoods, e.g., Sutherland et al. (2013).

16 Interviews are conducted face-to-face in the responding households’ homes.
